# Evidence-based interventions to reduce maternal malnutrition in low and middle-income countries: a systematic review

**DOI:** 10.3389/frhs.2023.1155928

**Published:** 2023-10-25

**Authors:** Shivani Shenoy, Priyanka Sharma, Aishwarya Rao, Nusrat Aparna, Deborah Adenikinju, Chukwuemeka Iloegbu, John Pateña, Dorice Vieira, Joyce Gyamfi, Emmanuel Peprah

**Affiliations:** ^1^ Department of Global and Enviromental Health, NYU School of Global Public Health, New York, NY, United States; ^2^ Global Health Program, Department of Social and Behavioral Sciences, ISEE Lab, NYU School of Global Public Health, New York, NY, United States; ^3^ NYU Health Sciences Library, NYU Grossman School of Medicine, New York, NY, United States

**Keywords:** maternal malnutrition, LMICs, implementation outcomes, evidence-based interventions, sustainability, adoption, cost-effectiveness, feasibility

## Abstract

**Introduction:**

Despite remarkable strides in global efforts to reduce maternal mortality, low-and middle-income countries (LMICs) continue to grapple with a disproportionate burden of maternal mortality, with malnutrition emerging as a significant contributing factor to this enduring challenge. Shockingly, malnourished women face a mortality risk that is twice as high as their well-nourished counterparts, and a staggering 95% of maternal deaths in 2020 occurred within LMICs. The critical importance of addressing maternal malnutrition in resource-constrained settings cannot be overstated, as compelling research studies have demonstrated that such efforts could potentially save thousands of lives. However, the landscape is marred by a scarcity of evidence-based interventions (EBIs) specifically tailored for pregnant individuals aimed at combatting maternal malnutrition and reducing mortality rates. It is against this backdrop that our study endeavors to dissect the feasibility, adoption, sustainability, and cost-effectiveness of EBIs designed to combat maternal malnutrition.

**Methods:**

Our comprehensive search encompassed eight prominent databases covering the period from 2003 to 2022 in LMICs. We began our study with a comprehensive search across multiple databases, yielding a total of 149 studies. From this initial pool, we eliminated duplicate entries and the remaining studies underwent a thorough screening process resulting in the identification of 63 full-text articles that aligned with our predefined inclusion criteria.

**Results:**

The meticulous full-text review left us with a core selection of six articles that shed light on interventions primarily centered around supplementation. They underscored a critical issue -the limited understanding of effective implementation in these countries, primarily attributed to inadequate monitoring and evaluation of interventions and insufficient training of healthcare professionals. Moreover, our findings emphasize the pivotal role of contextual factors, such as cultural nuances, public trust in healthcare, the prevalence of misinformation, and concerns regarding potential adverse effects of interventions, which profoundly influence the successful implementation of these programs.

**Discussion:**

While the EBIs have shown promise in reducing maternal malnutrition, their true potential for feasibility, adoption, cost-effectiveness, and sustainability hinges on their integration into comprehensive programs addressing broader issues like food insecurity and the prevention of both communicable and non-communicable diseases.

## Introduction

Maternal morbidity and mortality, particularly those stemming from preventable causes like malnutrition, continue to present formidable challenges in numerous countries. The early 2000s witnessed significant progress in enhancing the well-being of global populations, with the maternal mortality ratio (MMR) declining by 38% (1). Nonetheless, a comprehensive World Health Organization (WHO) report on maternal mortality trends has highlighted glaring disparities in maternal outcomes, influenced by a complex interplay of factors. These factors encompass malnutrition, poverty, socioeconomic status (SES), educational attainment, occupation, residential locality, and access to universal health coverage, among others (1).

Nutrition plays a pivotal role in shaping the physical well-being, mental health, and overall quality of life for expectant mothers and their offspring. Inadequate intake of essential macro- and micronutrients can have profound and lasting repercussions on one's health (2). During pregnancy, the absence of critical nutrients like zinc, calcium, folate, iodine, and iron can precipitate conditions such as preeclampsia, anemia, and hemorrhage, which sadly contribute to preventable maternal fatalities and childbirth complications that could otherwise be mitigated through nutritional interventions (3).

Conversely, an excess of nutrients, known as overnutrition, also imposes significant adverse health outcomes on mothers and expectant individuals, including the onset of gestational diabetes, obesity, pre-eclampsia, and cardiovascular disorders (4, 5). Given the detrimental effects of both undernutrition and overnutrition, our systematic review adopts a comprehensive perspective, encompassing overnutrition within the scope of malnutrition. This inclusive approach acknowledges that the adverse health consequences of malnutrition are amenable to intervention, offering the prospect of prevention and treatment. This, in turn, can yield positive outcomes for both the mother and the child, leading to a reduction in maternal morbidity and mortality (6).

According to the WHO report, a staggering 94% of all maternal deaths occurred in low and middle-income countries (LMICs), with approximately 810 deaths happening daily due to preventable pregnancy- and childbirth-related causes (1). Unfortunately, the progress in reducing maternal mortality in LMICs was significantly hampered by the redirection of workforce priorities and resources in response to the COVID-19 pandemic (7). Consequently, the pandemic exacerbated food insecurity by disrupting agricultural systems, and increasing poverty. Although maternal mortality rates were underreported during the pandemic, researchers estimate that over ten thousand maternal deaths were attributable to wasting (undernutrition) (8, 9). Specifically, in many overcrowded health facilities in LMICs, the pandemic intensified the burden on the maternal population as governments postponed and canceled services, including cesarean deliveries and nutritional programs, which had detrimental effects on maternal health outcomes (10).

Maternal morbidity and mortality associated with malnutrition are disproportionately high in LMICs such as Yemen, Somalia, South Sudan, and others (11, 12). In some of these nations, existing nutrition-based interventions are poised for scaling up to improve outcomes (13, 14). However, their sustainability is hindered by inadequate multi-sectoral partnerships, resource scarcity, and cost-prohibitive strategies (10). Many of these interventions, originally designed for populations in high-income countries, have not been adapted or adequately studied in LMICs due to limited funding (14, 15).

To comprehensively assess the existing literature on the implementation outcomes of evidence-based interventions (EBIs) targeting maternal malnutrition in LMICs, we conducted a meticulous systematic review. Our primary goal in this review is to scrutinize the feasibility, adoption, sustainability, and cost-effectiveness of EBIs aimed at mitigating maternal malnutrition.

We firmly believe that the insights derived from our analysis will prove invaluable to decision-makers at both local and national levels. In contrast, factors such as acceptability, appropriateness, fidelity, and penetration, though pertinent in many contexts, may carry lesser weight in the decision-making processes within LMICs. This assertion is corroborated by previous studies (16, 17), which underscore the critical importance of addressing these four specific outcomes when introducing interventions within communities to ensure their successful implementation.

This systematic review also holds the potential to fortify the World Health Organization's “Ending Preventable Maternal Mortality Strategy” (18), which seeks to curtail maternal mortality by furnishing member countries with evidence-based clinical and programmatic guidance. Furthermore, it aims to bolster research evidence available to stakeholders involved in the implementation of these strategies within the intended populations. In essence, our comprehensive review not only contributes to the broader academic discourse but also serves as a pragmatic resource for policymakers striving to combat maternal malnutrition in resource-constrained settings.

## Methods

### Search strategy

Our research endeavors encompassed a comprehensive literature search aimed at identifying research articles addressing maternal malnutrition within LMICs. Our meticulous search strategy incorporated specific keywords such as “maternal,” “malnutrition,” “feasibility,” “adoption,” “sustainability,” and “cost-effectiveness” in subject headings. Additionally, we utilized the World Bank's 2022 Country Classification as a vital filter criterion to delineate our list of LMICs (19). The intricate process of refining our search strategy was facilitated by the expertise of a dedicated librarian. Our quest for relevant literature led us to scrutinize several prominent databases, including PubMed, CINAHL, Cochrane, Google Scholar, Web of Science, EMBASE, and Global Health. This exhaustive systematic review spanned from October 2022 to January 2023, reflecting our commitment to thoroughness and comprehensiveness.

Moreover, our dedication to transparency is evident in the registration of our study on the Open Science Framework (OSF). This registration, completed on January 31, 2023, can be accessed at the following DOI: https://doi.org/10.17605/OSF.IO/7HN3T, ensuring accessibility and traceability of our research process.

### Inclusion/exclusion criteria

The search strategy adhered to the structured PICO format, encompassing Population, Intervention, Comparison, and Outcomes. Specifically, we focused on the population of interest—pregnant women residing in low- and middle-income countries (LMICs). Our primary area of investigation pertained to various treatments targeting malnutrition in this demographic. Notably, we did not establish a specific comparison group, as our emphasis lay in evaluating the implementation outcomes. In line with Proctor et al.'s seminal work (17), we honed in on four critical implementation outcomes: feasibility, sustainability, adoption, and cost-effectiveness. Our inclusivity criteria encompassed a diverse range of study designs, incorporating cross-sectional, longitudinal, cohort, retrospective, and ecological studies. Conversely, we judiciously excluded literature reviews, policy reports, and commentaries that did not directly address maternal malnutrition or were conducted in high-income countries. Furthermore, we imposed no restrictions regarding the age of the study population or the publication year, ensuring a comprehensive and unbiased examination of the available literature.

### Data extraction

Following our comprehensive literature search, we employed an organized workflow for efficient management. The retrieved articles were initially imported into EndNote and subsequently transferred to Covidence. Within this process, we meticulously addressed issues of duplication to ensure data integrity. To perform a rigorous evaluation of each article, two independent reviewers, NA and SS, methodically assessed their adherence to predefined criteria encompassing study design, methodology, population, interventions, and health outcomes. Any disparities or discrepancies in this screening process were thoughtfully deliberated upon and resolved through collaborative discussion between the two reviewers. Subsequently, we undertook an in-depth examination of the selected articles, extracting essential information such as study design, geographical context, sample size, interventions employed, and the various implementation outcomes under scrutiny. It is important to note that our guidance for outcome extraction was rooted in the definitions provided by Proctor et al. (17), ensuring a standardized and robust approach to evaluating implementation outcomes.

### Quality assessment

We rigorously evaluated the risk of bias and the overall quality of the included articles using the Mixed Methods Appraisal Tool (MMAT), as outlined in the work by Hong et al. (20). This comprehensive tool encompassed a set of screening questions applicable to all studies, with additional tailored questions for both qualitative studies and quantitative randomized controlled trials. To ensure a robust and impartial assessment, two independent reviewers conducted the critical appraisal of the selected studies, documenting their findings within the tool. Following this individual assessment, the researchers engaged in a constructive discussion to reconcile any disparities and ultimately generated a final quality assessment chart (see Table 1: Risk of Bias—MMAT).

**Table 1:** MMAT Risk of Bias assessment tool.

		Girard et al.	Heidkamp et al.	Makola et al.	Noznesky et al.	Ramakrishnan et al.	Saldanha et al.
Screening questions (for all types)	S1. Are there clear research questions?	Yes	Yes	Yes	Yes	Yes	Yes
	S2. Do the collected data allow to address the research questions?	Yes	Yes	Yes	Yes	Yes	Yes
Qualitative	1.1. Is the qualitative approach appropriate to answer the research question	Yes	Yes	NR	Yes	Yes	Yes
	1.2. Are the qualitative data collection methods adequate to address the research question?	Yes	No	NR	Not Applicable	Yes	Yes
	1.3. Are the findings adequately derived from the data?	Yes	Yes	NR	Yes	Yes	Yes
	1.4. Is the interpretation of results sufficiently substantiated by data?	Yes	Not Applicable	NR	Yes	Yes	Yes
	1.5. Is there coherence between qualitative data sources, collection, analysis and interpretation?	Yes	Not Applicable	NR	Yes	Yes	Yes
Quantitative randomized controlled trials	2.1. Is randomization appropriately performed?	NR	NR	Yes	NR	NR	NR
	2.2. Are the groups comparable at baseline?	NR	NR	Yes	NR	NR	NR
	2.3. Are there complete outcome data?	NR	NR	Yes	NR	NR	NR
	2.4. Are outcome assessors blinded to the intervention provided?	NR	NR	Not Applicable	NR	NR	NR
	2.5 Did the participants adhere to the assigned intervention?	NR	NR	Yes	NR	NR	NR
NR	NR	7	4	6	6	7	7

If included: Yes, if not included; No, if the assessment is not included, Not Applicable, if not required by the study; NR, if not reported; NR.

Furthermore, we diligently adhered to the Preferred Reporting Items for Systematic Reviews and Meta-Analysis (PRISMA) checklist, as recommended by Page et al. (21), in order to enhance the transparency and comprehensiveness of our study. This checklist guided us in ensuring the completeness and clarity of our reporting process.

## Results

**Figure 1:**
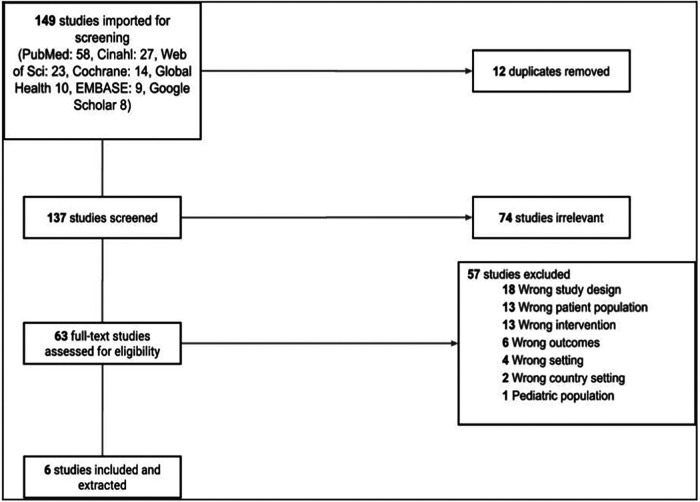
PRISMA Flowchart.

A comprehensive literature search, spanning six databases, yielded a total of 149 studies available for initial screening (distributed across databases as follows: PubMed = 58, CINAHL = 27, Web of Science = 23, Cochrane = 14, Global Health = 10, EMBASE = 9, and Google Scholar = 8). To ensure data integrity, we promptly eliminated 12 duplicates (as depicted in Figure 1). Subsequently, the independent screening of the remaining 137 studies by our team of reviewers culminated in the identification of 63 full-text articles. However, upon closer examination, the researchers unanimously deemed the majority of these studies irrelevant based on our predetermined exclusion criteria. Consequently, our full-text review was meticulously conducted for the 63 articles that initially met the inclusion criteria. Within this stage, we diligently assessed each article's adherence to crucial criteria, including study location within low- and middle-income countries (LMICs), relevance to maternal health, malnutrition, nutritional deficiency, and other pertinent factors. Regrettably, 41 articles were excluded, with reasons spanning misalignment with the designated patient populations, interventions, settings, study designs, outcomes, or even the country settings. The final selection of articles, however, authentically addressed evidence-based maternal nutrition interventions, health improvements, and child-care benefits within LMIC communities. For a detailed exposition of the characteristics and interventions found in these six articles, please refer to Table 2.

**Table 2:** Selected articles demographic characteristics and interventions.

Author	Year	Study design	Population	Country setting	Intervention
Girard et al.	(22)	Qualitative study	Maternal population of Taraba region	Northeastern Nigeria	IFA supplementation
Heidkamp et al.	(10)	Qualitative study	RMNCH	LMICs- Senegal, Burkina Faso, Ehtiopia, Zambia Tanzania, Rwanda, Nepal Bangladesh, India, Vietnam Kyrgyzstan, Peru	IFA and calcium supplementation Agriculture programs Social protection programs (vouchers, food subsidies) Nutrition counseling
Makola et al.	(23)	Randomized, placebo-controlled double-blind effectiveness trial	Pregnant women	Tanzania	Micronutrient-fortified beverage
Noznesky et al.	(24)	Qualitative study	Women and adolescent girls	Bihar, India	IFA supplementation
Ramakrishnan et al.	(25)	Qualitative study	Maternal population of Taraba region	Tamil Nadu, Uttar Pradesh—India	IFA supplementation Education programs Take -home food rations
Saldanha et al.	(26)	Qualitative study	Pregnant women	Tigray, SNNPR Ethiopia	IFA supplementation Productive safety net program Targeted supplementary food

The systematic review encompassed six distinct studies conducted across four LMICs. Two of these studies were situated in India, with one each in Nigeria, Tanzania, and Ethiopia. Notably, one study, led by Heidkamp et al., spanned multiple sites, encompassing Ethiopia, Zambia, Tanzania, Rwanda, Nepal, Bangladesh, India, and Peru. The predominant study design featured in our review was qualitative research, with five out of the six studies conducting interviews with key informants in either hospital or community settings. The studies varied in duration, ranging from 2 months to 5 years. Though no age limit was defined, the participants primarily were of reproductive age.

We subjected the included studies to a comprehensive assessment, focusing on key implementation outcomes, namely feasibility, adoption, sustainability, and cost-effectiveness. Notably, five of the reviewed studies provided insights into both feasibility and cost-effectiveness, while all six studies contributed to our understanding of adoption and sustainability.

For a more detailed breakdown of the specific implementation outcomes addressed in these six articles, please refer to Table 3. Furthermore, to gain a deeper understanding of the intended target population, intervention components, and the resultant service outcomes, Table 4 provides a comprehensive overview of these essential study details.

**Table 3:** Implementation outcomes.

Author	Feasibility	Adoption	Sustainability	Cost-effectiveness
Girard et al.	x	–	x	x
Heidkamp et al.	x	x	x	x
Makola et al.	–	x	x	–
Noznesky et al.	x	x	x	x
Ramakrishnan et al.	x	x	x	x
Saldanha et al.	x	x	x	x

**Table 4:** Characteristics of interventions and service outcomes.

Author	Priority issues included	Intervention components	Service outcomes
Girard et al. (22)	• Anemia in Northeast Nigeria • Maternal nutritional status before pregnancy—[prepregnant body mass index (BMI) and short stature] and during pregnancy (weight gain) in Taraba State, Nigeria	• Iron–folic acid (IFA) • Supplementation • Fortification of staples with micronutrients (Salt iodization) • Anthelmintics • Antimalarial drugs	• 55.7% women consumed any IFA during pregnancy • Less than 20% consumed recommended 90 tablets during pregnancy • 50.5% households in Taraba state using iodized salts • Less than 6% of women in Taraba reported consuming anthelmintic agents during their last pregnancy • Less than 4% women used an insecticide -treated bednet; 20% reported receiving malaria medications during their last pregnancy
Heidkamp et al. (10)	• Global undernutrition in non-pregnant adolescents and women of reproductive age • Global maternal malnutrition during pregnancy	• Micronutrient intervention • IFA Supplementation • Calcium supplementation • Nutritional counseling • Antenatal care • Balanced energy protein supplements	• National programs for IFA exist in Ethiopia, Kenya, Senegal, Tanzania and India • Guidance exists but delivery platform needs to be identified for adolescents • Maternal micronutrient supplements are more cost-effective than IFA • Calcium pills more expensive than IFA and multiple micronutrient supplements • Nutrition counseling improves diet and supplement adherence, food security in pregnant women • Balanced energy protein supplements show evidence of improved birth outcomes in undernourished women. It is feasible but data on cost is limited
Makola et al. (23)	• Prevention of iron deficiency • Reduce anemia • Improve hemoglobin concentration for pregnant women in Tanzania	• Micronutrient-fortified beverage containing 11 micronutrients (iron, iodine, zinc, vitamin A, vitamin C, niacin, riboflavin, folate, vitamin B-12, vitamin B-6 and vitamin E)	• 4.16 g/L increase in hemoglobin concentration • 3 g/L increase in ferritin • Reduced risk of anemia by 51% • Reduced risk of iron deficiency anemia by 56% • The risk of iron deficiency reduced by 70% among those who had iron deficiency at baseline • The risk of iron deficiency was reduced by 92% among those who had adequate stores
Noznesky et al. (24)	• Improving maternal outcomes in Bihar, India	• IFA, Calcium fortification and other key interventions and delivery platforms to address maternal undernutrition • Barriers and innovations for delivering interventions	• Shortage of personnel and lack of incentivization to deliver interventions • Low prioritization of interventions to improve maternal nutrition • IFA supplementation best for all women of reproductive age (regardless of pregnancy status) • Strengthen systems for monitoring and evaluation and engage more community health workers • Strengthen policies that prioritize maternal nutrition • Multisectorial partnerships with women's organizations and community level leadership
Ramakrishnan et al. (25)	• Improving maternal outcomes in Tamil Nadu and Uttar Pradesh, India	• Micronutrient supplementation • Food-based strategies • Growth monitoring • Education and counseling	• Prioritize maternal nutrition in national programs with clear targets for LBW and teenage pregnancies • Strategies to improve women's education and status, delayed childbirth • Improving monitoring and evaluation in existing programs • Support for R&D in anemia • Improved multisectoral partnerships
Saldanha et al. (26)	• Maternal undernutrition in Ethiopia • Maternal anemia • Maternal thinness and stunting • Intrauterine growth retardation (IUGR)	• Use of supplement and drugs during pregnancy • Iodized salt use • Anemia	• Community-based health services as a platform for supplementation, social and behavior change communication • Increased M&E • Incentivization to delay childbirth • Engage more CHWs in anemia prevention • Incorporation of social and behavior change communication for maternal nutrition programs • Link nutrient fortification programs to programs targeting food insecurity

### Feasibility

The assessment of feasibility within these studies revolved around the potential for scaling the intervention to encompass other regions within the country (22, 24, 25). Additionally, the evaluation of intervention feasibility encompassed practical considerations such as addressing logistical challenges within the supply chain.

In Nigeria, Ethiopia, and India, the assessment of the feasibility of evidence-based interventions (EBIs) was conducted through qualitative interviews directly involving both the maternal population and local-level implementers (22, 25, 26). However, it's noteworthy that the concept of feasibility was not explicitly addressed in the context of Tanzania. Participants mentioned that the supply of nutritional supplements varied as per funding, quantity available, and delivery methods. They stated that often times the supply would be delayed by weeks which deprived the expecting mothers of the much-needed nutritional supplements.

### Adoption

Consequently, the concept of adoption in these studies encompassed two key facets: first, it delved into the provider's inclination or preference to embrace the intervention, and second, it examined the extent to which mothers adhered to the intervention, including their consumption of supplements or participation in follow-up activities. Notably, all of the reviewed articles extensively reported on the aspect of adoption concerning EBIs. In the case of India and Nigeria, local-level implementers mentioned that adoption was improved when the dietary education and supplements were provided by their local women-led centers (24, 26). These accounts also shed light on the nuanced challenges encountered within various regions of these countries. For instance, the barriers to adoption in the Tigray region vary from that in the Southern Nations, Nationalities and Peoples Region (SNNPR) in Ethiopia and can be attributed to religious beliefs and dietary practices (26). Participants in SNNPR more frequently reported fasting during pregnancy to keep their digestive tract clean whereas participants in Tigray reported fasting during religious occasions. These beliefs hinder the adoption of supplementation and the community workers are not trained to break such taboos (26).

Within the context of Tanzania, an intervention involving a fortified nutrient beverage demonstrated enhanced outcomes and a high degree of adoption, particularly when presented in beverage form (23). However, it is important to note that the reports did not explicitly confirm the adoption as a singular intervention as the fortified beverages were provided to women who were visiting antenatal centers and receiving other supplementations. Across all studies, a recurrent theme emerged- maternal nutritional interventions are coupled with complementary interventions such as malaria treatments, antihelminth supplements, Water, Sanitation, and Hygiene programs due to resource constraints.

### Sustainability

Sustainability of the intervention, in this context, was gauged based on the intervention's potential to be extended and continued over time under government auspices. Across all articles, sustainability was qualitatively addressed and some of the key barriers stated by the participants were the inconsistency in leadership and funding (10, 24, 26). Due to the resource-limited settings, the community workers were overworked and underpaid which led to high staff turnover and nobody to continue the intervention. Additionally, the lack of a fixed budget for maternal nutrition hindered the successful sustainability of the program. Notably, all the articles underscored the importance of fostering sustainability through collaborative efforts involving local and national policymakers, along with strategic partnerships with other programs, including initiatives related to malaria and school-based meal programs. These collaborations were seen as instrumental in ensuring the lasting impact of the interventions.

Furthermore, all the articles emphasized the critical need for enhanced monitoring and evaluation mechanisms, which were consistently cited as one of the most significant barriers to assessing sustainability.

### Cost-effectiveness

Finally, the evaluation of the intervention's cost-effectiveness hinged on whether the studies delved into the financial aspects of implementing the intervention as it relates to the health benefits of the intervention. This included an exploration of whether the costs were covered by government agencies, non-governmental organizations (NGOs), international organizations, or if they were anticipated to become out-of-pocket expenses for mothers following the intervention.

Cost-effectiveness findings were predominantly conveyed qualitatively, spanning individual, local, and national levels (10, 26). Notably, the discussions highlighted that iron-folic acid (IFA) and calcium supplementation proved to be cost-effective, especially with the support and endorsement of policymakers (23).

### Risk of bias assessment

We employed the Mixed Methods Appraisal Tool (MMAT) to rigorously assess the quality of the included studies (20). The evaluation criteria centered on the approach, data collection methods, and the findings derived from each study. The collective quality of the articles garnered a favorable assessment, with six out of seven articles achieving scores of 83% or higher on the MMAT (as outlined in Table 1: Risk of Bias—MMAT). Furthermore, it's noteworthy that the overall risk of bias across all studies was deemed to be low. However, it's essential to acknowledge the possibility of both sampling and reporting bias, given that the key informants interviewed were affiliated with their respective countries' ministries of health.

## Discussion

While the EBIs utilized in the studies reviewed effectively mitigate maternal malnutrition, their successful implementation in LMICs necessitates consideration of various factors. Our analysis of six studies demonstrates that these EBIs, when integrated into existing programs, exhibit feasibility, adoption, cost-effectiveness, and sustainability. Our systematic review unveiled a notable paucity of data concerning maternal health outcomes within LMICs. This scarcity was underscored by the inclusion of merely six articles meeting our search criteria. Nevertheless, these articles provided valuable insights into service delivery barriers, facilitators, and the prospective implementation of interventions.

Within these articles, crucial observations and recurrent themes were meticulously documented, serving as a critical foundation for identifying prevailing gaps in the delivery of interventions aimed at mitigating maternal malnutrition in LMICs. It is imperative to emphasize that a comprehensive understanding and proactive addressing of these themes have the potential to catalyze a significant and transformative impact on the scalability and effectiveness of maternal health interventions.

### Low prioritization of maternal nutrition by governments

The reviewed articles shed light on a pervasive issue: the low prioritization of maternal nutrition at both local/state and national levels. This systemic shortcoming served as an underlying barrier to effectively addressing maternal malnutrition. This lack of prioritization manifested in several ways, including inadequate funding, limited planning, budget allocation, and restricted access to services for the intended population.

Furthermore, a recurring theme across the articles was the dearth of partnerships among health promoters, governments, and non-governmental organizations (NGOs). This absence of collaboration hindered the allocation of resources, hindered access to services, and limited awareness of maternal nutrition services within communities.

Supply chain issues emerged as a significant challenge, with Tanzania, Ethiopia, and India, among other LMICs, grappling with these logistical hurdles, often as a consequence of limited budgets and poor planning. For instance, in Nigeria's Taraba state, supply chain issues led to unequal access to free iron-folic acid (IFA) supplements [as discussed by (22)]. This resulted in low utilization of this highly acceptable and cost-effective intervention, as the medicine was accessible at no cost in some areas while costing US $1.2 for a two-week supply in others, posing a financial barrier for many pregnant women.

Additionally, a recurrent barrier highlighted in the studies was the shortage of essential resources, including weighing scales, laboratory equipment, and healthcare workers. These inadequately equipped health facilities eroded trust within the community, ultimately leading to reduced adoption of available nutrition programs by pregnant women.

### Food insecurity and misinformation

When addressing malnutrition in LMICs, it is imperative to consider the significant impact of food insecurity and misinformation. Studies conducted in India have unveiled a multitude of barriers that hinder the scalability of effective interventions, notably influenced by socioeconomic factors such as poverty, discrimination, and low health literacy among females [as elucidated by (25)].

Misinformation within certain communities has prompted pregnant women to consume smaller portions in an attempt to maintain lower body weights and avoid weight gain during pregnancy. Financial constraints often lead some women to sacrifice their own nutritional needs to accommodate other family members, resulting in anemia and stunting among adolescent females. The lack of information regarding the causes and prevention of anemia stands as a pivotal factor contributing to its high prevalence among women [as reported by (22)]. This underscores the urgent need for counseling and awareness programs that not only elucidate the etiology and consequences of anemia but also address prevalent misperceptions and cost barriers that drive communities toward traditional healing approaches.

To enhance the feasibility and adoption of nutrition-based interventions, there are promising avenues to explore, notably in the realm of social and behavior change communication. Establishing partnerships with women-centered NGOs and organizations dedicated to combating food insecurity, coupled with initiatives promoting female literacy, can prove to be potent allies. These partnerships offer robust service delivery platforms for counseling, education, awareness campaigns, and supplement distribution to women of reproductive age.

Practical strategies, such as leveraging local schools and available technology, offer tangible delivery platforms for not only supplements but also nutrition counseling, family planning services, and anemia prevention initiatives. Engaging youth groups and the adolescent population in advocacy and education campaigns can further amplify the impact of these services and contribute significantly to the achievement of global nutrition targets [s demonstrated by (10)].

Furthermore, it is worth noting that nutrition counseling has demonstrated a tangible impact on increasing adherence to interventions like IFA and multiple micronutrient supplementation, underscoring its role as a sustainable approach to addressing maternal malnutrition.

### Cost-effectiveness of micronutrient supplementation and multi-sectoral partnerships

Research indicates that the cost-effectiveness of interventions is enhanced when multiple micronutrient supplements are employed, surpassing the efficacy of singular approaches like calcium or IFA supplements [as highlighted in (27)]. Given that iron deficiency and other micronutrient deficiencies seldom occur in isolation among pregnant women, the comprehensive utilization of multiple micronutrient supplements, in conjunction with nourishing foods and beverages, emerges as an effective strategy to mitigate the risks associated with nutrient deficiencies and anemia during pregnancy. The introduction of these multifaceted interventions not only bolsters their acceptability but also enhances their adoption and sustainability when integrated within the framework of antenatal care clinics [as discussed in (23)]. Consequently, the successful implementation of nutrition-based interventions necessitates a holistic approach that comprehensively addresses both the direct and indirect outcomes related to nutrition.

For instance, the delivery of supplemental packaged protein bars, enriched micronutrients, and IFA supplements, in tandem with anthelmintics and malaria treatment, emerged as popular interventions. Establishing partnerships with programs dedicated to combating malaria, helminth infections, water, sanitation, and hygiene (WASH), poverty reduction, and other related initiatives can serve as cost-effective service delivery strategies for governments and local agencies. This collaborative approach not only optimizes resource utilization but also affords the intended population access to a wide spectrum of essential services concurrently [as exemplified in (26)].

### Monitoring & evaluation of existing programs

A recurring obstacle often cited in the literature is the insufficient awareness of supplementation guidelines among healthcare workers, as extensively noted in Saldanha et al. (26). For instance, in Ethiopia, key informants pointed to the scarcity of IFA supplements as a reason behind the inefficient distribution of these vital resources. Furthermore, they raised concerns about supplements not being dispensed and subsequently expiring at community centers.

A common thread running through all the studies centered on the pervasive lack of monitoring and evaluation (M&E) in existing workforce and program structures. The absence of systematic M&E within current programs hampers decision-makers' ability to gain valuable insights into the barriers and facilitators of targeted EBIs, which is important for prioritizing effective budget allocation, especially in resource-constrained settings.

## Strengths and limitations

This systematic review boasts several strengths, including its comprehensive assessment of both qualitative and quantitative literature. The rigorous and exhaustive search encompassed peer-reviewed sources, enhancing the reliability of the findings. While the primary target population was women of reproductive age in LMICs, it is worth noting that numerous resource-scarce communities in middle and high-income countries have similarly implemented programs aimed at addressing service delivery gaps. Consequently, conducting periodic, in-depth assessments of these interventions holds potential benefits for advancing their scalability. Such assessments can help identify specific implementation outcomes and ascertain whether resolving challenges within these communities is feasible, supported by evidence-based data.

However, a limitation of this review pertains to its exclusive focus on maternal malnutrition outcomes, which effectively restricted the scope of the research. As a result, only one of the six reviewed articles was published in 2021, significantly curtailing the ability to assess the latest data and comprehend the pandemic's impact on maternal malnutrition in LMICs. The inclusion of more recent literature could provide valuable insights for the timely analysis of barriers to intervention implementation and better inform decision-making aimed at mitigating maternal malnutrition.

## Conclusion

While the EBIs examined in our study prove effective in mitigating maternal malnutrition, it is imperative to recognize that their successful implementation hinges on multiple considerations, particularly within the context of LMICs. Among the six studies we reviewed, we observed that EBIs aimed at reducing maternal malnutrition can indeed be feasible, adoptable, cost-effective, and sustainable when integrated into existing programs. Nonetheless, the paucity of available literature underscores the urgent need for a call to action to foster more research endeavors. Such research should aim to provide solutions that bolster delivery platforms, foster multi-sectoral partnerships, and identify funding opportunities for the robust monitoring and evaluation of implemented EBIs.

Expanding on our findings, we emphasize that to curtail the prevalence of malnutrition-related maternal morbidity and mortality in LMICs, it is imperative for local and national-level decision-makers to prioritize comprehensive approaches. Collaboration with community organizations through multi-sectoral partnerships emerges as a pivotal strategy. This holistic approach should encompass critical components such as food provisions and nutritional supplementation, integrated within programs designed to address food insecurity, and the prevention of both communicable and non-communicable diseases.

Furthermore, our review underscores the inefficacy of independent implementation of single micronutrient interventions like IFA or calcium supplementation. Such approaches are neither cost-effective, sustainable, nor comprehensive in addressing the broader issue of undernutrition. Disparities in access to these interventions are often rooted in inadequate funding and a dearth of rigorous monitoring mechanisms. The absence of standardized guidelines for healthcare workers further exacerbates the overall lack of effective intervention adoption.

To remedy these challenges, it is imperative to allocate sufficient funding and resources while providing comprehensive training to community healthcare workers. This ensures a consistent supply of essential resources crucial for maintaining the quality of proposed EBIs. These strategies collectively form a critical toolkit for ensuring quality improvement at every stage of intervention implementation to improve maternal health outcomes and prolonged sustainability of these evidence-based interventions.

Looking ahead, future studies focusing on LMICs should actively engage local stakeholders to solicit systematic input, garner consistent support, and facilitate policy development. The adoption of implementation practice strategies to assess desired outcomes is a vital step in advancing the effectiveness of these interventions.

## Data Availability

The original contributions presented in the study are included in the article/Supplementary Material, further inquiries can be directed to the corresponding author.

## Appendix: World Bank 2022: Low and Middle Income Country List

(“LMICs” [tw] OR “LMIC” [tw] OR “developing countries” [tw] OR “developing country” [tw] OR “low income countries” [tw] OR “low income country” [tw] OR “middle income countries” [tw] OR “middle income country” [tw] OR “Global South” [tw] OR “resource poor” [tw] OR “low resource” [tw] Afghan OR Afghani OR Afghanistan OR Albania OR Albanian OR Algeria OR Algerian OR Angola OR Angolan OR Argentina OR Armenia OR Armenian OR Azerbaijan OR Bangladesh OR Bangladeshi OR Benin OR Beninese OR Belize OR Belizean OR Bhutan OR Bhutanese OR Bolivia OR Bolivian OR Burkina Faso OR Burkinabe OR Burundi OR Burundian OR Belarus OR Belarusian OR Bosnia OR Bosnian OR Botswana OR Brazil OR Brazilian OR Cambodia OR Cambodian OR Central African Republic OR Central African OR Chad OR Chadian OR Comoros OR Comoran OR Congo OR Congolese OR Chile OR Chilean OR China OR Chinese OR Colombia OR Columbian OR Costa Rica OR Costa Rican Eritrea OR Eritrean OR Ethiopia OR Ethiopian OR Gambia OR Gambian OR Guinea OR Guinean OR Haiti OR Haitian OR Kenya OR Kenyan OR Korea OR Korean OR Kyrgyz OR Kyrgyzstan OR Liberia OR Liberian OR Madagascar OR Malagasy OR Malawi OR Malawian OR Mali OR Malian OR Mozambique OR Mozambican OR Myanmar OR Myanmarese OR Burmese OR Nepal OR Nepalese OR Niger OR Nigerian OR Rwanda OR Rwandan OR Sierra Leone OR Sierra Leonean OR Somalia OR Somalian OR Tajikistan OR Tajik OR Tadzhik OR Tanzania OR Tanzanian OR Togo OR Togolese OR Uganda OR Ugandan OR Zimbabwe OR Zimbabwean OR Cameroon OR Cameroonian OR Cape Verde OR Cape Verdian OR Cape Verdean OR Cote d'Ivoire OR Ivory Coast Ivorian OR Djibouti OR Egypt OR Egyptian OR El Salvador OR Salvadorian OR Salvadorans OR Fiji OR Fijian OR Georgia OR Georgian OR Ghana OR Ghanaian OR Guatemala OR Guatemalan OR Guyana OR Guyanese OR Honduras OR Honduran OR Indonesia OR Indonesian OR India OR Indian OR Iraq OR Iraqi OR Kiribati OR Kosovo OR Kosovar OR Laos OR Lao OR Laotian OR Lesotho OR Marshall Islands OR Marshallese OR Mauritania OR Mauritanian OR Micronesia OR Micronesian OR Moldova OR Moldovan OR Mongolia OR Mongolian OR Morocco OR Moroccan OR Nicaragua OR Nicaraguan OR Nigeria OR Pakistan OR Pakistani OR Papua New Guinea OR Papua New Guinean OR Paraguay OR Paraguayan OR Philippines OR Filipino OR Samoa OR Samoan OR “Sao Tome and Principe” OR “Sao Tome and Principe” OR Senegal OR Senegalese OR Solomon Islands OR Solomon Islander OR Sri Lanka OR Sri Lankan OR Sudan OR Sudanese OR Eswatini OR Swazi OR Swaziland OR Syria OR Syrian OR East Timor OR East Timorese OR Tonga OR Tongan OR Turkmenistan OR Turkmen OR Tuvalu OR Tuvaluan OR Ukraine OR Ukrainian OR Uzbekistan OR Uzbek OR Vanuatu OR Vietnam OR OR Dominica OR Dominican OR Ecuador OR Ecuadorean OR Gabon OR Gabonese OR Grenada OR Grenadian OR Iran OR Iranian OR Jamaica OR Jamaican OR Jordan OR Jordanian OR Kazakhstan OR Kazakhstani OR Latvia OR Latvian OR Lebanon OR Lebanese OR Libya OR Libyan OR Macedonia OR Macedonian OR Malaysia OR Malaysian OR Maldives OR Maldivian OR Mauritius OR Mauritian OR Mexico OR Mexican OR Montenegro OR Montenegrin OR Namibia OR Namibian OR Palau OR Palauan OR Peru OR Peruvian OR Russia OR Russian OR Serbia OR Serbian OR South Africa OR South African OR Saint Lucia OR Saint Vincent OR Suriname OR Surinamer OR Thailand OR Thai OR Tunisian OR Turkey OR Uruguay OR Venezuela OR Venezuelan OR Vietnamese OR West Bank OR Gaza OR Yemen OR Yemeni OR Yemenite OR Zambia OR Zambian).
